# Industrial hemp (*Cannabis sativa* L.)—a valuable alternative crop for growing in agricultural soils contaminated with heavy metals

**DOI:** 10.1007/s11356-023-30474-z

**Published:** 2023-10-26

**Authors:** Marko Flajšman, Katarina Košmelj, Helena Grčman, Darja Kocjan Ačko, Marko Zupan

**Affiliations:** Department of Agronomy, Biotechnical Faculty, University of Ljubljana, Jamnikarjeva, 101 1000 Ljubljana, Slovenia

**Keywords:** Industrial hemp, Heavy metals, Phytoremediation, Fibers, Seed, Yield

## Abstract

Hemp (*Cannabis sativa* L.) is a multiuse plant, which has been abundantly studied for phytoremediation purposes in recent years. The majority of experiments were performed in greenhouses with potted plants where hemp showed promising results. Only few studies tested hemp on site in heavy metal–polluted agricultural soil in real environmental conditions and practical assessments of hemp phytoremediation feasibility are lacking. We conducted a comprehensive study using 2 legal industrial hemp varieties (Futura 75 and Tisza) at three differently polluted locations (heavily polluted location, HP; moderately polluted location, MP; and slightly polluted location, SP) in the heavy metal contaminated Celje valley in Slovenia and determined the content of Pb, Zn, and Cd in 5 plant organs/tissues. The yield of each organ/tissue was determined as well to enable us to calculate the phytoremediation potential (PP). On average, plants grown in the HP location accumulated the highest values of all examined elements, followed by plants from the MP location and plants from the SP location, showing that the content of heavy metals in soil influences the accumulation in plants. Accumulation of Pb/Zn/Cd by plant organs/tissues was distributed in the following order: inflorescences (Pb-4.10/Zn-92.8/Cd-0.50 mg/kg) > seeds (Pb-1.79/Zn-92.6/Cd-0.27 mg/kg) > roots (Pb-1.15/Zn-15.0/Cd-0.44 mg/kg) > stem bark (Pb-0.42/Zn-12.4/Cd-0.23 mg/kg) > stem woody core (Pb-0.34/Zn-4.6/Cd-0.15 mg/kg). The only exception was for Cd, where roots accumulated a higher value than seed, yet lower than inflorescences. PP was calculated by multiplying hemp tissue/organ yield by the relative concentrations of heavy metal. The highest PP for Pb and Cd were achieved at the HP location (3.80 and 0.23 g/ha/vegetation period). On the other hand, tissue/organ yield was more important for high PP of Zn, where the SP location reached the highest PP for Zn (148.5 g/ha/vegetation period) due to the highest yields. Only seeds from HP and MP locations accumulated a too high content of Pb; otherwise, all other fibers and seeds can be safely used in the textile and food industry. Results of this study showed that hemp cannot be considered an efficient plant for the phytomanagement of contaminated areas. Nevertheless, hemp cultivation in heavy metal–polluted agricultural soils seems feasible since the majority of tissues/organs were not contaminated and different products can be obtained from various parts of the hemp plant.

## Introduction

Two of the most lethal inorganic contaminants of water and soil are potentially toxic metals and metaloides, known as heavy metals. This group encompasses As, Cd, Pb, Hg, Cr, Co, Cu, Ni, Zn, Se, and U (Kanwar et al. 2020). Some of those elements are essential for plant nutrition (like Cu and Zn); however, excessive concentration in soil could have a negative effect on plant growth (Ali et al. 2019; Kabata-Pendias 2011). Although naturally occurring heavy metals that enriched soils have been found all over the world (Adriano 1986), most often soil pollution is a man-made problem (Ernst 1996). Industrial activities (e.g., electronics, automobile, thermal power plants), mining, transportation, sewage disposal, e-wastes, and agrochemicals are the main anthropogenic sources releasing toxic heavy metals (Alloway 2012; Kumar et al. 2017a; Ali et al. 2019; Vardhan et al. 2019; Wu et al. 2019).

Contamination of soil with heavy metals poses great risks to living organisms, both flora and fauna (Ekmekyapar et al. 2012). Agricultural sustainability is in serious jeopardy when crops are cultivated in polluted areas (Lone et al. 2008). Elevated concentrations of heavy metals in soil can decrease microbial biodiversity and negatively affect its activity, which leads to deteriorated soil fertility (Xu et al. 2018). Such low soil health status affects agricultural productivity, causes the loss of agricultural yields, and decreases the quality of the food grown (Popescu et al. 2009; He et al. 2019). Furthermore, soil heavy metal runoff contaminates surface and ground water (Vries et al. 2007). Finally, when heavy metals enter the food chain via feed or food, they could negatively affect human health and cause several fatal diseases (Liu et al. 2013; Chu et al. 2019; Ali et al. 2019).

There are several approaches to cleaning up contaminated soil. An eco-friendly and sustainable technique for the elimination of heavy metals from soil in an in situ manner is phytoremediation. This is a “green technique,” where plants or microorganisms are used in order to reduce the level of hazardous chemicals in water, air, and soil environments by remediation, uptake, removal, extraction, stabilization, immobilization, destruction, and sequestration of contaminants (Kanwar et al. 2020; Shah and Daverey 2020). Hyperaccumulators are plants capable of tolerating and accumulating high levels of metals. These are usually annual herbs, with low or no economic value and very little biomass (i.e., *Thlaspi caerulescens*, *Minuartia verna*, *Ipomea alpina*), but can achieve high metal concentrations in above-ground tissues (Brooks and Robinson 1998). However, for efficient phytoremediation, high biomass is also needed. Since phytoremediation is a time-consuming process, the best choice is to employ crops of commercial interest in phytoremediation activities (i.e., maize and sunflower) or fast-growing shrubs and trees (i.e., willows and poplars) (Khan et al. 2000; Vassilev et al. 2004).

Hemp (*Cannabis sativa* L.) is an alternative crop, whose cultivation is more environmentally friendly than traditional crops (Fike 2016; Żuk-Gołaszewska and Gołaszewski 2018). Being a low-input crop, hemp needs low fertilization rates and rare use of plant protection products and is capable of high weed suppression due to its fast growth (Bócsa and Karus 1998; van der Werf 2004; Sandler and Gibson 2019). Furthermore, it fits very well into crop rotation schemes (Desanlis et al. 2013). With low environmental impact and well-known techniques of cultivation, hemp demonstrates very suitable characteristics to fit perfectly into a sustainable agricultural system (van der Werf 2004; Żuk-Gołaszewska and Gołaszewski 2020).

Hemp has been abundantly studied for phytoremediation purposes for two main reasons: first is its biological characteristics; hemp is an annual crop, which produces massive above-ground biomass and it has been shown in many studies that absorption of heavy metals into green organs is not negligible (Ahmad et al. 2016; Rheay et al. 2020), thus being capable of removing quite a substantial amount of heavy metals from polluted soil. The plant has a deep taproot with a high capability to absorb and to some extent also accumulate heavy metals like lead, nickel, cadmium, zinc, and chromium (Linger et al. 2002; Citterio et al. 2005; Ahmad et al. 2016). Due to a short life cycle, other crops can be grown in the same growing season in the same field. The second reason is its high commercial value, since the global market for hemp consists of more than 25,000 products including submarkets for textiles, agriculture, automotive, food and beverages, paper, furniture, construction, recycling, and personal care (Salentijn et al. 2015; Crini et al. 2020). However, earlier studies have shown that all parts of hemp plants contain heavy metals (McPartland and McKernan 2017) and this is why their use as a commercially utilizable plant material is limited. Especially high levels of heavy metals in seed, leaves, and fiber would disqualify hemp from being used in traditional industries, e.g., food and textiles (Linger et al. 2002; Mihoc et al. 2013).

Hemp cannot be marked as a hyperaccumulator (De Vos et al. 2022); however, accumulation of heavy metals in some plant organs could exceed limit values and therefore cannot be used in traditional ways. Besides, controversial information about which plant parts accumulate the highest content of some heavy metals exists in the literature. Most phytoremediation research has been conducted with potted plants in controlled greenhouse conditions. To the best of our knowledge, only a few studies more than two decades ago performed experiments in real field conditions using hemp for phytoremediation of heavy metals from polluted soil (Linger et al. 2002; Angelova et al. 2004; Di Candilo et al. 2004) and lately, no new detailed on-site studies have tried to address issues related to managing soil pollution in natural conditions in real-world situations. Such studies are even more important if contaminated areas are arable land, where crops for food are grown. Therefore, the aims of our research were to (i) compare the accumulation of three heavy metals (Pb, Zn, and Cd) in 5 different plant organs/tissues of 2 hemp varieties grown at three differently polluted agricultural locations in the Celje valley in Slovenia; (ii) compare the phytoremediation potential of plant organs/tissues based on their yields obtained from on-site field experiments; and (iii) assess whether some plant organs/tissues grown in Pb-, Zn-, and Cd-polluted soils are suitable to be used for food or textiles.

## Materials and methods

### Experimental site design

**Fig. 1:**
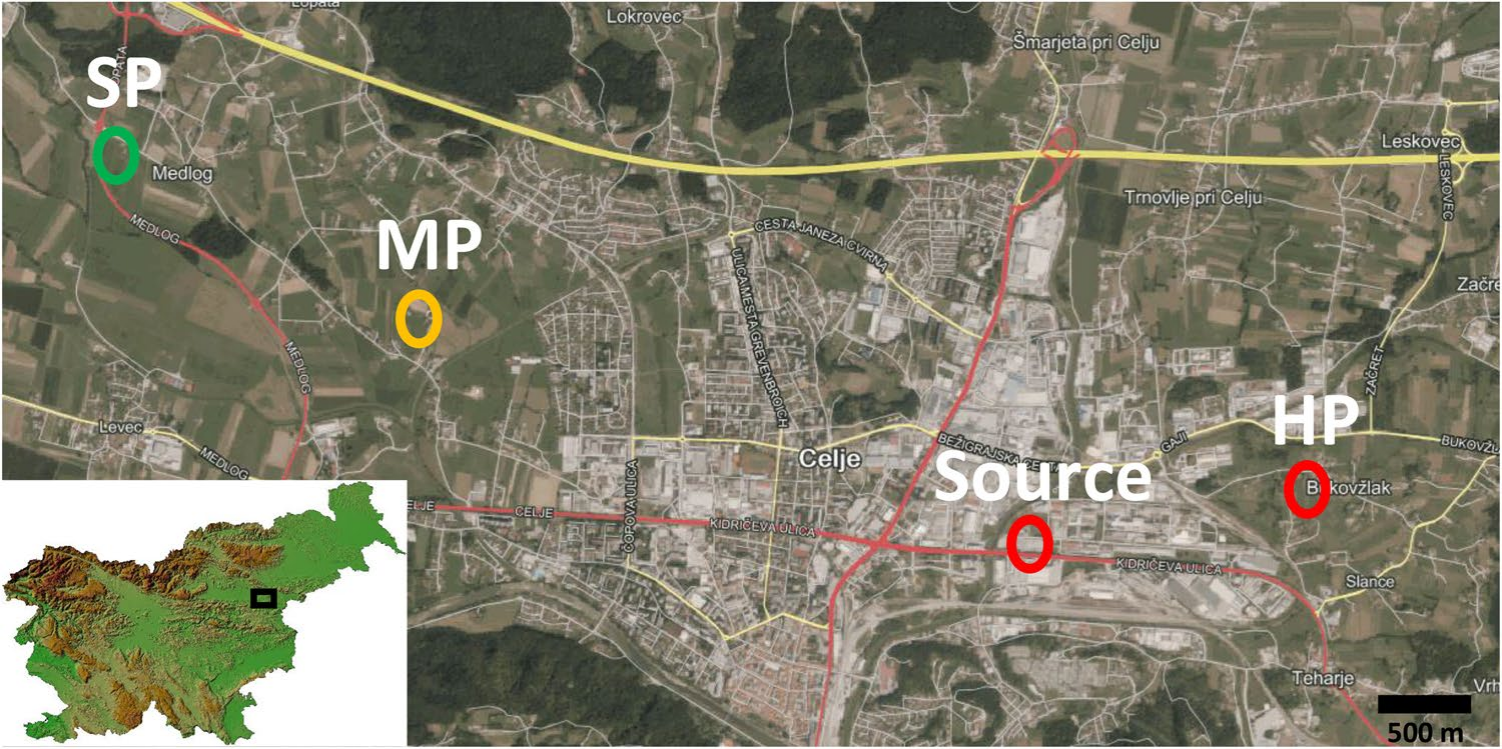
Map of the experimental plots in Celje valley (HP, heavily polluted location; MP, moderately polluted location; SP, slightly polluted control location; source: “Cinkarna Celje” zinc factory, the source of heavy metal pollution)

The field experiment was conducted in the growing season of 2017 in the Celje valley. The area of Celje is contaminated with many potentially toxic metals due to zinc ore smelting; Cd concentration in the soil on more than 4000 ha exceeds the warning value for Cd (2 mg/kg) and more than 50 ha has Cd content over a critical value (12 mg/kg). Zn and Pb are also potentially toxic metals present in larger quantities in this area. The experimental plots were situated in working farmers’ fields, where crops for food and feed are grown. The experimental plots were chosen at different distances from the source of pollution, which is the zinc factory “Cinkarna Celje.” The first plot was chosen very close (approx. 1.7 km) to the pollution source and was named as heavily polluted (HP). The second plot was situated approx. 3.4 km from the pollution source and was moderately polluted (MP). The last location was 5.3 km away from the source of pollution and served as a slightly polluted (SP) control location (Fig. 1).

At each location, two varieties of hemp were grown, namely a monoecious variety Futura 75 and a dioecious variety Tisza. Sowing took place on 12 May 2017 with a sowing density of 300 seeds/m^2^ and a row distance of 12.5 cm using a Wintersteiger plot seeder. The experiment was designed as a randomized complete block experiment. The basic plot size was 6 × 3 m (18 m^2^) with four replications per variety (total 144 m^2^). The composite soil sample was taken at each location; the 30 soil increments were randomly taken at the whole area from plowing depth (0–20 cm) using a soil probe. During the growing season, no fertilizers, no herbicides, no fungicides, and no insecticides were used.

### Sampling of hemp plants

Harvest was carried out manually on the 11th and 13th of October 2017. Only the inner 4 square meters of each basic plot were considered for different sampling, and each single square meter was labelled A, B, C, and D. From square meter A, plants were counted and divided by sex (only for the dioecious variety Tisza). Furthermore, 25 plants per gender were randomly chosen for height determination and, after drying at 105°C until constant weight, stems were used for heavy metal determination. Plants from square meter B were pulled out with the roots and the roots were used for further analysis. Plants from the whole four square meters were used for the determination of the fresh above-ground weight of the hemp plants and after the removal of the inflorescences, the weight of fresh stems was determined. Inflorescences were dried, and after mechanical seed removal, the weight of dry inflorescences and the weight of the seed were determined. From all this data, the different yields per hectare were calculated. Dry tissues were used for the measurement of heavy metal content. Phytoremediation potential (PP) was calculated as the product of tissue/organ content (mg/kg) multiplied by tissue/organ yield (kg/ha). Total phytoremediation potential for each variety was determined as the sum of the average PP of all organs/tissues. Graphs, showing averages with standard errors, were drawn using the “ggplot2” package in the statistical software program R version 3.2.5 (R Core Team 2019).

### Preparation of plant tissues for heavy metal content determination

Five different plant organs/tissues were prepared for heavy metal content determination, namely stems (divided into the bark and woody core), roots, inflorescences, and seeds. Approx. 5 to 10 g of dry samples was prepared. Stems were prepared as follows: the bottom 10 cm of each stem was discarded in order to avoid contamination with the possible residues in the soil. Then, the next 30 cm of the stems was manually divided into the bark (where the fibers are located) and woody core or hurd. Twenty-five stems were combined in one sample. For the dioecious variety Tisza, stem samples were additionally separated by gender into male and female plants. Roots were prepared by thoroughly washing them in distilled water and cut into small pieces using a ceramic knife. All the samples were fine ground into pieces smaller than 2 mm using a rotary mill Retsch ZM 10. Inflorescences were prepared by grinding manually and using a 5-mm and 2-mm sieve to remove soil and rocks. Seeds were milled using a Retsch MM200.

### Analytical methods

One composite soil sample and three plant samples of each tissue and variety of hemp per site were analyzed. The laboratory measurement uncertainty was used for the error assessment of soil analytical parameters. Pretreatment of soil samples (ISO 11464; 2006) and basic soil properties were determined in the Centre for Soil and Environmental Science analytical laboratory at the Biotechnical faculty. The soil pH was determined in extraction with CaCl_2_ solution according to ISO 10390 (2006); the method of dry combustion was used to evaluate the content of organic matter (ISO 10694 1996); and the ammonium lactate solution (AL method) was used for extraction of plant available phosphorous (P_2_O_5_) and potassium (K_2_O), detection of phosphorous was done with a spectrophotometer, detection of potassium with atomic absorption spectrophotometer.

The measurement of Pb, Zn, and Cd content in soil from the experimental location was performed in the laboratory of Bureau Veritas Minerals (Vancouver, Canada) using inductively coupled plasma mass spectrometry (ICP-MS) after aqua regia extraction (ISO 11466 1996). The same laboratory also measured the concentration of the same heavy metals in different plant organs/tissues in three replications per variety per location using the same analytical method.

### Weather conditions

**Fig. 2:**
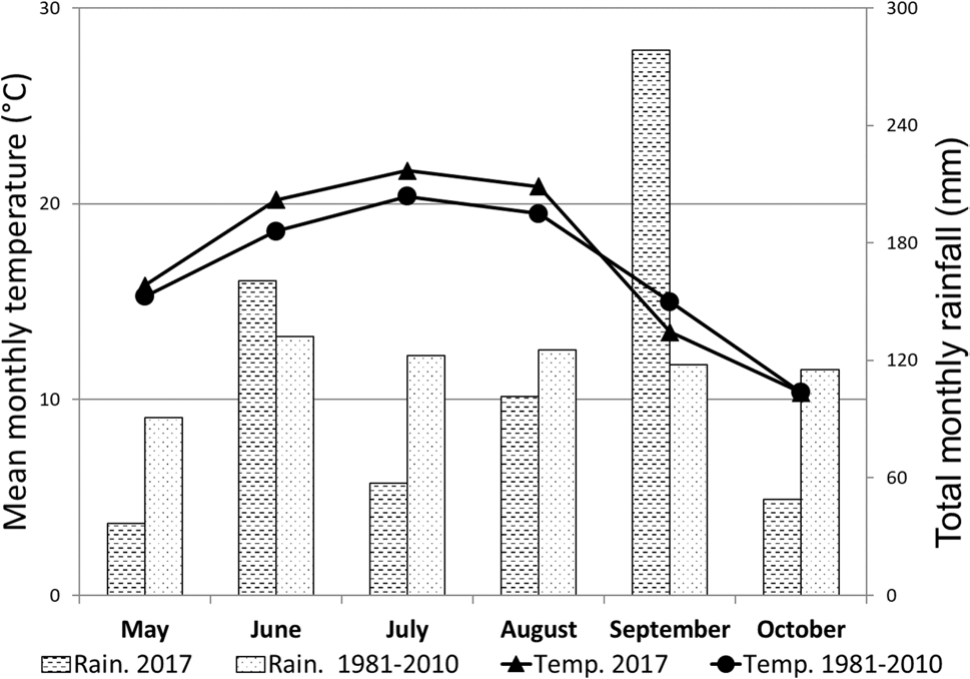
Weather conditions (mean monthly temperature and total monthly precipitation) during the growing season of 2017 and the long-term average (1981–2010)

In 2017, the mean monthly temperature was higher than the 30-year average (1981–2010) with the exception of September, when the temperature was 1.6°C lower (Fig. 2). This month also had excessive rainfall (278 mm), when the amount of rain was 2.4 times higher than the long-term average. The rainfall in June was also above the long-term average; otherwise, other months had less precipitation. At the location HP, hail at the beginning of June damaged the plants, but they recovered and continued to grow.

## Results and discussion

### Chemical characteristics and heavy metal content of the soil in the test locations

Chemical properties of the soil at each test site and the concentration of potentially toxic metals Pb, Zn, and Cd are shown in Table 1.

**Table 1:** Soil properties and content of Pb, Zn, and Cd in the upper 20 cm at the test sites, and normative values according to Slovenian legislation

Location	Distance from pollution source (km)	pH in CaCl_2_	P_2_O_5_ (mg/100 g soil)	K_2_O (mg/100 g soil)	Organic matter (%)	Pb (mg/kg soil)	Zn (mg/kg soil)	Cd (mg/kg soil)
Field experiments at Celje valley								
HP	1.7	7.0 ± 0.3	26.0 ± 2.6	72.5 ± 7.3	4.9 ± 0.8	98.2 ± 15.7	708 ± 113.3	6.6 ± 1.1
MP	3.4	6.8 ± 0.3	20.2 ± 2.0	29.8 ± 3.0	5.0 ± 0.9	83.7 ± 13.4	227 ± 36.6	1.7 ± 0.3
SP	5.3	6.2 ± 0.2	18.3 ± 1.8	24.1 ± 2.4	5.3 ± 0.9	41.4 ± 6.6	157 ± 25.1	1.1 ± 0.2
Slovenian normative immission values								
Critical value						530	720	12
Alert value						100	300	2
Limit value						85	200	1

*HP*, heavily polluted location; *MP*, moderately polluted location; *SP*, slightly polluted control location; critical, alert, and limit values are immission values for heavy metal contamination in soils in Slovenia (Decree on limit values, alert thresholds and critical levels of dangerous substances in the soil 1996). Measure of variability for chemical properties of the soil shows measurement uncertainty for analytical laboratory

As expected, the nearest location (HP) contained the highest values of all three measured heavy metals, where the content of Zn exceeded the critical value, the contents of Cd heavily exceeded the alert value, and the content of Pb was in the range of the alert value. In addition, the soil in this location was extremely rich in potassium but exhibited the lowest value of organic matter. The slightly polluted control location (SP) which was located furthest from the source of pollution showed the lowest concentration of all heavy metals (below the limit values, only Cd was in the range of the limit value) and the highest organic matter content (Table 1).

### The height of the plants and the yields of plant tissues/organs

**Fig. 3:**
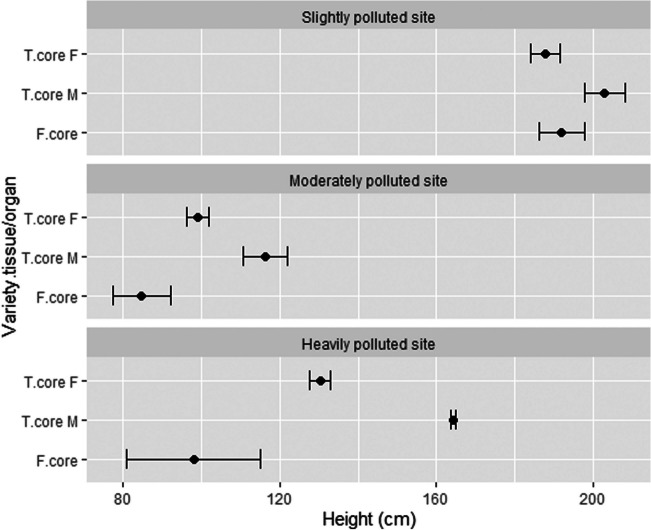
The height of the plants of two varieties at three testing locations. T, Tisza; F, Futura 75; F, female; M, male; *n* = 4

At the location SP, the plants reached the greatest height, showing the best growing conditions. The HP site showed somehow limited growth, but lower plants can be attributed to the hail in June, which damaged the tips of the plants and slowed the growth. The lowest plants were measured at the MP site (Fig. 3), very likely due to massive soil compaction, which was observed at harvest when plants were pulled out with the roots, which grew only into the top 10 cm layer of the soil. The hemp plant can reach up to 5 m in height (Small 2015). Plant height is not only genotype dependent, but rather soil and climatic characteristics, together with agro-techniques, play an important role in plant agronomic performance (Desanlis et al. 2013). Therefore, plant height is a very usable indicator for the evaluation of growing conditions.

**Fig. 4:**
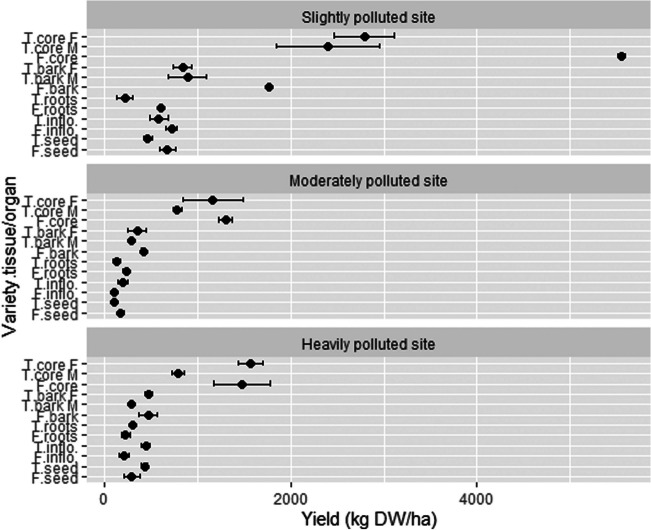
Yields (kg/ha) of different hemp tissues/organs at three locations. T, Tisza; F, Futura 75; F, female; M, male; inflo, inflorescences; DW, dry weight; *n* = 4

In this experiment, yields of different plant parts were evaluated (Fig. 4). As expected, stems represented the major share (79.5%) among the yields (combined for both varieties and all locations) and the woody core represented the majority of the stem yield (75.5%) and the rest was assigned to the bark. The yield of dry stems (combining woody core and bark) was the highest at the location SP and reached 7.3 t/ha for the variety Futura 75 and 6.9 t/ha for the variety Tisza. This is in the range of usual dry stem yield reached in some other Middle European countries (Tang et al. 2016; Flajšman and Ačko 2020). Yields of stems at location MP were the lowest (1.7 t/ha for Futura 75 and 2.6 t/ha for Tisza). The highest yield of dry roots was achieved by the variety Futura 75 at location HP (0.6 t/ha). Since roots were pulled out, fine roots broke off and remained in the soil. Therefore, root yield in this study represents only the mass of taproots. Our result is comparable with the roots yield from Amaducci et al. (2008), who noticed a slightly higher taproot yield (1.0–1.4 t/ha) probably because of better growth, since their above-ground biomass was also higher. The yield of inflorescences was the highest for Futura 75 at SP (0.72 t/ha) and the lowest for the same variety at location MP (0.11 t/ha). The seed yield was similar to the European average value (0.67 t/ha in the year 2018; Faostat 2020) at location SP (an average of 0.57 t/ha for both varieties), where the HP and MP locations exhibited substantially lower seed yields.

### The concentration of Pb in different hemp tissues/organs and phytoremediation potential

**Fig. 5:**
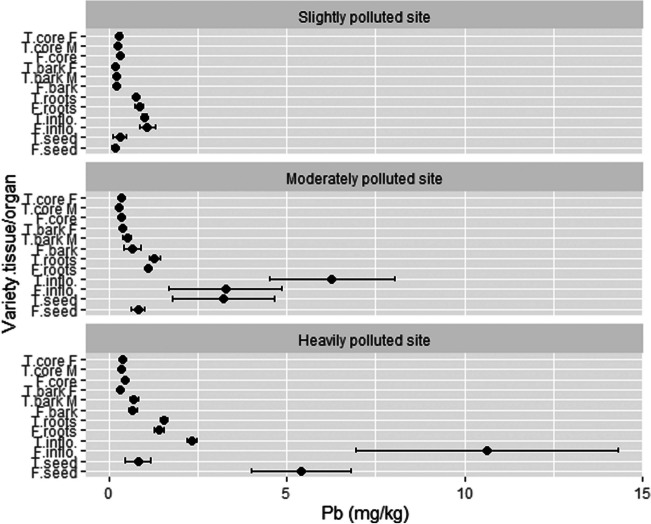
The concentration of Pb in different hemp tissues/organs. T, Tisza; F, Futura 75; F, female; M, male; inflo, inflorescences; *n* = 3

At location SP, the soil content of Pb was the lowest compared to that at the other two locations. At SP, roots and inflorescences of both varieties accumulated the highest values of Pb (from 0.74 up to 1.08 mg/kg) among all plant parts. At the MP location, the highest content of Pb was determined in inflorescences of the Tisza variety (6.27 mg/kg), followed by inflorescences of Futura 75 (3.27 mg/kg) and seeds of the Tisza variety (3.23 mg/kg). Roots of both varieties also showed little increased content of Pb (up to 1.28 mg/kg). At location HP, inflorescences (10.64 mg/kg) and seeds (5.40 mg/kg) of Futura 75 showed the highest concentration of Pb. Again, increased content of Pb was observed in roots (up to 1.53 mg/kg) and also in stem bark (up to 0.70 mg/kg) of Tisza (Fig. 5). The accumulation of Pb in organs/tissues follows in this order: inflorescences (4.10 mg/kg) > seeds (1.79 mg/kg) > roots (1.15 mg/kg) > stem bark (0.42 mg/kg) > stem woody core (0.34 mg/kg). Interestingly, the female bark of Tisza exhibited the lowest Pb concentration compared to male and monoecious stem bark. On the other hand, the male woody core of Tisza showed the lowest Pb concentration compared to female and monoecious stem woody core. Regarding test locations, the average content of Pb in hemp plants follows HP (2.08 mg/kg) > MP (1.54 mg/kg) > SP (0.46 mg/kg). The variety Futura 75 (1.82 mg/kg) accumulated 1.8 times higher concentration of Pb than Tisza (1.03 mg/kg).

Linger et al. (2002) noticed the highest accumulation of Pb in hemp leaves (22.4 mg/kg) (variety USO 31), followed by fibers and hurds. In seeds, they noticed the lowest concentration, yet the same as in our experiment (1.8 mg/kg). The soil in their experiment contained a much higher amount of Pb (454 ppm). Ahmad et al. (2016) also detected a high accumulation of Pb in hemp leaves (39 mg/kg). Di Candilo et al. (2004) measured similar content of Pb in roots (up to 1.88 mg/kg), but lower in the stem as in this study. On the other hand, Angelova et al. (2004), who grew hemp at polluted and non-polluted locations, noticed a higher amount of Pb in the roots (38.2 mg/kg), probably because of a much higher content of Pb in the soil at the polluted location (200.3 mg/kg). In their study, Pb content decreased in the following order: roots > stems > leaves > seed > fiber.

In Commission Regulation (EC) No 1881/2006, the maximum level of Pb in oil (foodstuff) is set at 0.1 mg/kg and for seed (if hemp seed is placed in group 3.1.12) at 0.2 mg/kg of wet weight. Even though, in our experiment, the concentration of Pb in hemp seed is expressed by dry weight, the concentrations in seeds from HP and MP are much higher than the described maximum levels, meaning that only seeds from SP could be used for food production. By the Oeko-tex Standard 100 (Oeko-Tex-Initiative 2021), the highest total content of Pb in textile materials and products is set at 90 mg/kg. In our study, stem bark (where fibers are deposited) contains from 0.17 to 0.70 mg/kg. Although we have not isolated pure fibers for Pb analysis, hemp bark is mainly composed of fibers (fiber represents approx. 70% of bark according to Mediavilla et al. 2001); therefore, we can conclude that fibers from our analyzed stem bark could be safely used in the textile industry.

**Fig. 6:**
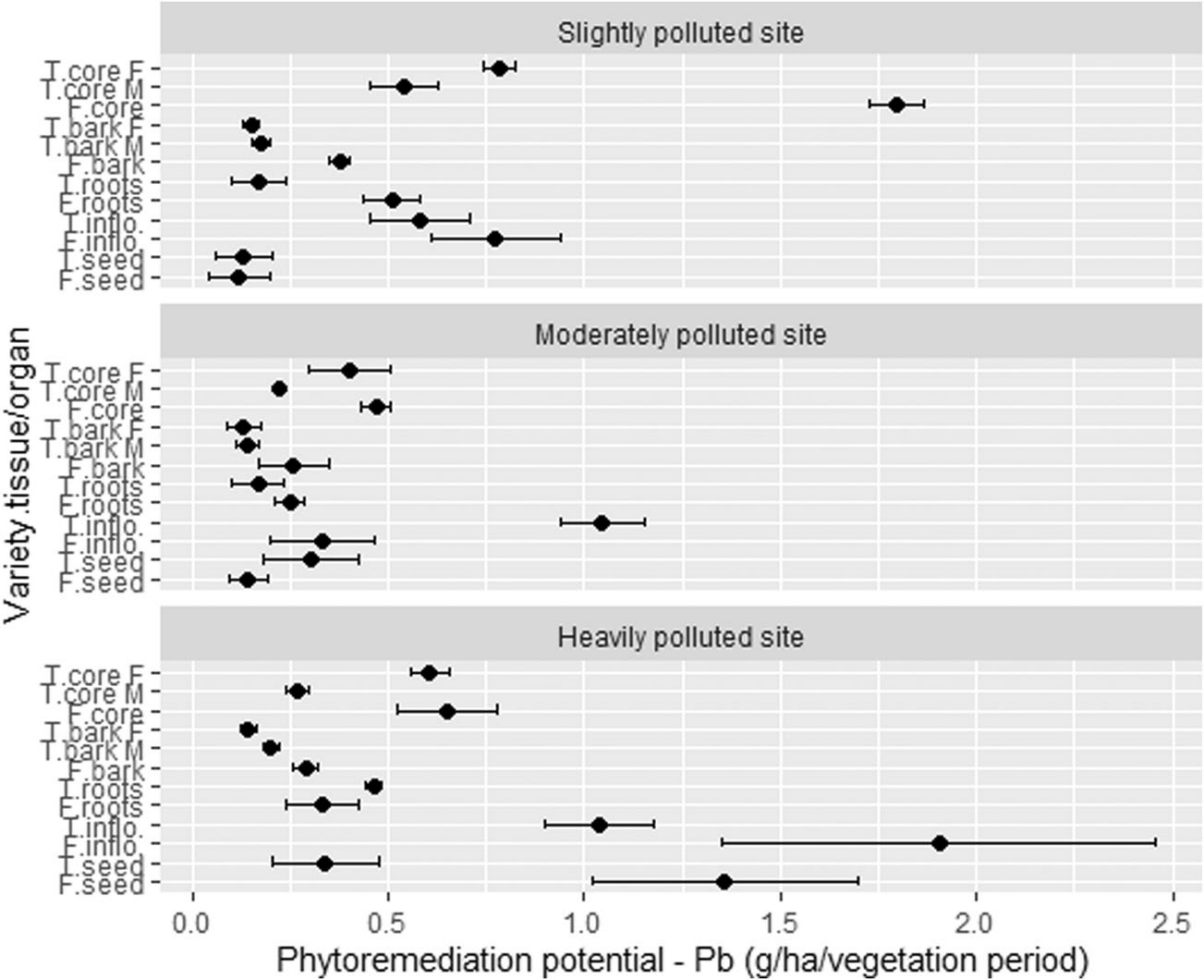
The phytoremediation potential for Pb of different hemp tissues/organs. T, Tisza; F, Futura 75; F, female; M, male; inflo, inflorescences; *n* = 3

Phytoremediation potential (PP) was calculated by multiplying hemp tissue/organ yield by the relative concentrations of heavy metal in this tissue/organ. PP for Pb by variety tissue/organ is shown in Fig. 6. At SP, the highest PP was achieved in the stem woody core of Futura 75 variety (1.80 g Pb/ha), followed by the stem woody core of Tisza (1.32 g Pb/ha combined for male and female stem woody core). Inflorescences of both varieties also accumulated higher values at this location (0.77 g Pb/ha for Futura 75 and 0.58 g Pb/ha for Tisza). At location MP, inflorescences of Tisza took the highest amount of Pb (1.05 g/ha), followed by woody cores of Tisza (0.62 g/ha combined for male and female) and Futura 75 (0.47 g/ha). At the HP location, inflorescences of Futura 75 accumulated 1.9 g Pb/ha and seeds of the same variety 1.36 g Pb/ha. Next were inflorescences of Tisza with 1.04 g Pb/ha, followed by woody cores of Tisza (0.87 g/ha combined for male and female stem woody core) and woody core of Futura 75 (0.65 g/ha). On average, stem woody core extracted the highest amount of Pb (0.97 g/ha), followed by inflorescences (0.95 g/ha) and seeds (0.40 g/ha). Total PP for the varieties Futura 75 and Tisza are as follows: 3.57 and 2.53 g/ha (average 3.05 g/ha) for SP, 1.45 and 2.41 g/ha (average 1.93 g/ha) for MP, and 4.53 and 3.06 g/ha (average 3.80 g/ha) for the HP site.

PP is heavily influenced by crop yield. Although the SP location had a much lower content of Pb in the soil and consequently plants extracted had a lower content of Pb in their organs/tissues, yields (especially of stem woody core) were the highest, resulting in higher PP compared to the location MP, where soil was more polluted, but crop yields were low. At the HP location, the highest PP was achieved, on average 3.80 g Pb/ha. Di Candilo et al. (2004) noticed a much higher uptake of Pb from artificially polluted soil by two hemp varieties, which was up to 11.35 g Pb/ha/year. Although the content of Pb in measured plant parts was slightly lower compared to our study, they achieved much higher crop yields resulting in higher PP.

These are, however, very modest PP outcomes since some other plants can achieve much higher uptakes, e.g., maize and vetiver were capable of accumulating 758 g/ha and ≈ 800 g/ha, respectively, and when citric acid was added, up to 11 times increase in the ability to remove Pb from the soil was observed (Freitas et al. 2013). However, the content of Pb in the soil in the study of Freitas et al. (2013) was 1850 mg/kg, which is approx. 20–45 times higher than in our study. *C. sativa* is also capable of tolerating a very high concentration of Pb (up to 1600 mg/kg; our unpublished results from pot experiments), and in such growing conditions, higher PP of hemp is also expected. Very obviously the concentration of Pb in the soil has a tremendous impact on the metal uptake by the plant (see the “General discussion” section for an explanation).

### The concentration of Zn in different hemp tissues/organs and phytoremediation potential

**Fig. 7:**
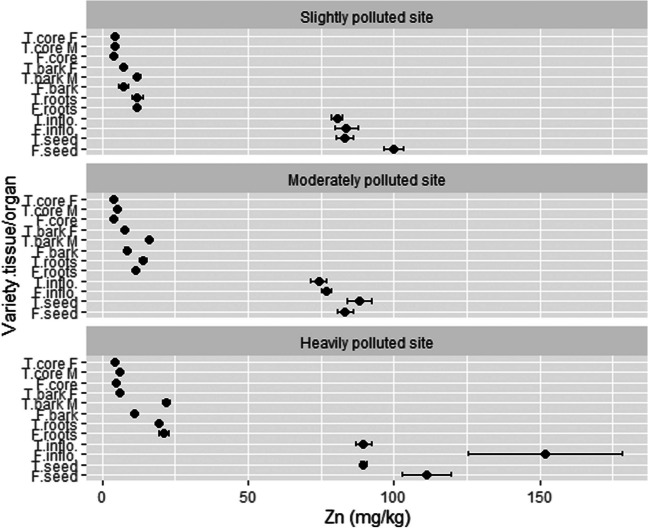
The concentration of Zn in different hemp tissues/organs. T, Tisza; F, Futura 75; F, female; M, male; inflo, inflorescences; *n* = 3

The highest concentration of Zn at the SP location was detected in seeds (99.8 mg/kg) and inflorescences (83.8 mg/kg) of the variety Futura 75, followed by seeds (83.2 mg/kg) and inflorescences (80.4 mg/kg) of the variety Tisza (Fig. 7). Roots (on average 11.9 mg/kg) and bark (on average 8.8 mg/kg) show little Zn accumulation, while under 4.5 mg/kg was detected in stem woody core. A similar trend regarding tissue/organ concentration was observed at the other two locations; at MP, generative organs of both varieties accumulate the highest values of Zn, where the average value for seed was 85.8 mg/kg and for inflorescences 75.5 mg/kg. Stem woody core accumulated the lowest value of Zn (on average 4.3 mg/kg). At HP, the variety Futura 75 again accumulated the highest content in inflorescences and seeds (151.9 and 111.3 mg/kg), whereas Tisza showed about 89.6 mg/kg in both generative organs. Roots (20.4 mg/kg) and bark (13.0 mg/kg) showed lower values, yet higher than at SP and MP locations. Again, stem woody core contained the lowest value (5.1 mg/kg). Organ/tissue Zn accumulation was inflorescences (92.8 mg/kg) > seeds (92.6 mg/kg) > male stem bark (16.8 mg/kg) > roots (15.0 mg/kg) > female/monoecious stem bark (8.0 mg/kg) > stem woody core (4.6 mg/kg). Intriguingly, the male bark of Tisza contains on average 2.1 times higher concentration of Zn compared to the female stem bark of Tisza or monoecious Futura 75. The average content of Zn in hemp plants by location is as follows: HP (44.8 mg/kg) > SP (34.2 mg/kg) > MP (32.8 mg/kg) and the average concentration of Zn in the variety Futura 75 was 46.1 mg/kg and 31.0 mg/kg in variety Tisza.

Our results show that hemp plants had accumulated a much higher amount of Zn in their generative parts (inflorescences and seed) compared to vegetative organs (roots and stems). This observation supports the research by Malik et al. (2010), who found out that the translocation factor value of *C. sativa* for Zn is above 1, meaning that the accumulation of metal Zn in shoots is high. In their in situ experiment in a polluted industrial zone in Pakistan, the concentration of Zn in hemp shoots was 43.9 mg/kg, compared to 27 mg/kg in roots. On the other hand, Praspaliauskas et al. (2020) found out that Zn was equally distributed among the leaves, stems, and roots in a pot experiment where sewage sludge and sewage sludge char were added as soil amendment for hemp cultivation. Approximately equal Zn distribution by plant parts was noticed also by Angelova et al. (2004), yet flowers (78.6 mg/kg) and seeds (73.5 mg/kg) grown in a higher polluted location (536.1 mg Zn/kg of soil in top 20 cm) accumulated the highest amount of Zn, compared to roots, stems, and fiber. Ahmad et al. (2016) detected only 4.5 mg/kg in hemp leaves. Zielonka et al. (2020) observed an 11.95% increase in Zn content in generative organs of three hemp varieties when sewage sludge (as fertilizer) was added to the soil. In our study, we noticed a more than doubling (27.5%) in the generative organs of two hemp varieties at the HP location compared to the SP location.

Special attention should be paid to hemp seed used in the food industry. Zinc is a ubiquitous and essential element, necessary for maintaining biochemical and physiological functions in the human body (Radwan and Salama 2006). Therefore, the content of Zn in foodstuff is not officially regulated. But there are some limit values proposed to be taken into consideration to protect humans from harmful effects. According to INCHEM (International Programme on Chemical Safety) by WHO and EPA (U.S. Environmental Protection Agency), dietary zinc intakes could be around 20 mg per day for 70-kg adults (INCHEM 2021; EPA 2002). For our study, this means that around 200 g of seed from the HP site could be consumed daily and up to 15% more from the two other less Zn-polluted locations. Mihoc et al. (2013) found that Zn accumulation is up to 3 times larger in hulled hempseed core compared to hempseed husk, meaning that more care should be taken when consuming only hulled hempseeds.

Zn is not considered a toxic element in textile production and a value for total content is not mentioned among the toxic elements in the list of the Oeko-tex Standard 100 (Oeko-Tex-Initiative 2021). However, the Standard sets a limit in Annex 6 of extractable zinc of 750 mg/kg in textile materials and products. This is on average more than 72 times higher content as was measured in our study in the bark of plants, where fibers are located. Therefore, Zn poses no threat to human health if hemp fibers from this study were used for the production of textiles.

**Fig. 8:**
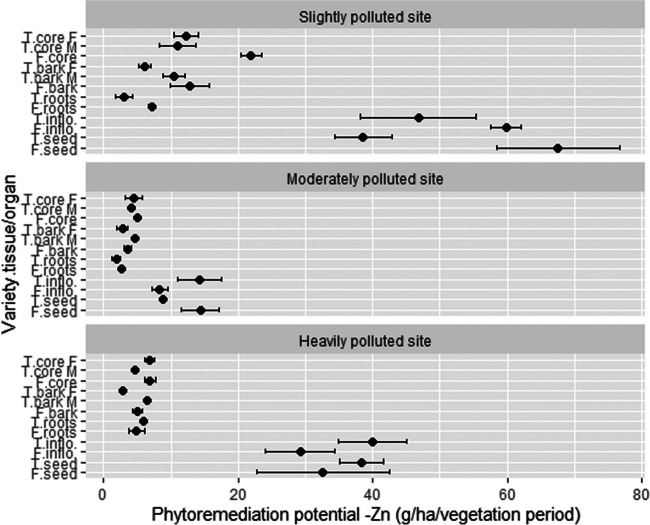
The phytoremediation potential for Zn of different hemp tissues/organs. T, Tisza; F, Futura 75; F, female; M, male; inflo, inflorescences; *n* = 3

Due to the high concentration of Zn in generative organs, inflorescences and seeds had the highest phytoremediation potential for Zn at every location. The highest PP was observed for Futura 75 seed (67.6 g Zn/ha) and inflorescences (59.8 g Zn/ha) at SP, followed by inflorescences (46.8 g Zn/ha) and seed (38.5 g Zn/ha) of Tisza. Due to the high yield of the core, this tissue also exhibited increased PP, 23.2 g Zn/ha for the woody stem core of Tisza and 21.9 g Zn/ha for Futura 75. MP showed lower PP values where seeds and inflorescences reached 14.4 g Zn/ha and 8.3 g Zn/ha for Futura 75 and 14.2. g Zn/ha and 8.8 g Zn/ha for Tisza. Overall, the seed (39.9 g Zn/ha) and inflorescences (38.3 g Zn/ha) of Tisza outperformed the seed (32.6 g Zn/ha) and inflorescences (29.2 g Zn/ha) of Futura 75. Other tissues/organs exhibited on average more than 6 times lower PP values. Interestingly, roots were the hemp plant organ with the lowest PP (up to 5.9 g Zn/ha) (Fig. 8). Ranking hemp tissues/organs regarding PP is as follows: seed (33.4 g Zn/ha) > inflorescences (33.0 g Zn/ha) > stem woody core (12.8 g Zn/ha) > bark (9.1 g Zn/ha) > roots (4.2 g Zn/ha). Futura 75 and Tisza achieved total PP of 169.1 and 127.9 g/ha (average 148.5 g/ha) for SP, 33.7 and 40.8 g/ha (average 37.3 g/ha) for MP, and 78.6 and 104.8 g/ha (average 91.7 g/ha) for the HP site.

It is interesting that the SP location achieved the highest PP for Zn, although the soil concentration of Zn was 4.5 times lower than at the HP location. From the results of the plant Zn distribution, it is evident that test location had a low influence on the differences among Zn accumulation in various hemp plant parts. A different trend was observed by Angelova et al. (2004), who detected considerably lower concentrations of zinc in all plant parts at the control location, which contained only 32.9 mg Zn/kg in soil, compared to a polluted location, which exhibited 14.7 times higher Zn soil concentration. In our study, the biomass yields of plant parts differed considerably between locations and obviously this was the main factor influencing the PP of Zn. Our results showed that to achieve high phytoremediation efficiency of Zn, biomass yield is a more important variable than the plant’s accumulation. This was not the case for some other crops. In a long-term field experiment with artificially polluted soil using up to 1550 mg/kg of zinc sulfate, Wenger et al. (2002) determined that *Nicotiana tabacum* and *Zea mays* are capable of absorbing up to 11,100 g/ha and 13,500 g/ha, respectively. The very high PP of these crops is the product of the high yield of biomass (e.g., up to 20.4 t/ha of dry weight for *Z. mays*) and high biomass concentration (e.g., up to 1905 mg/kg in *N. tabacum*). Zn uptake was high also because of the presence of sulfur, which acts as a chelator agent and enhances the zinc solubility and uptake (Kayser et al. 2000). Using chelates also improved Zn uptake in hemp; Kos and Leštan (2004) added an EDDS chelator to the contaminated soil in a pot experiment (1300 mg Zn/kg soil) and PP rose to 8257 g Zn/ha. Kos et al. (2003) determined PP 2630 g Zn/ha with the addition of EDTA chelate and 3680 g Zn/ha with the addition of EDDS.

### The concentration of Cd in different hemp tissues/organs and phytoremediation potential

**Fig. 9:**
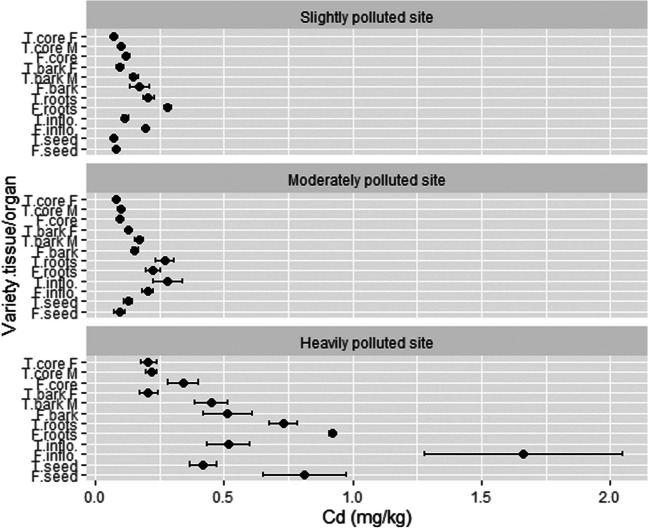
The concentration of Cd in different hemp tissues/organs. T, Tisza; F, Futura 75; F, female; M, male; inflo, inflorescences; *n* = 3

The concentration of Cd in different plant tissues/organs was generally low (Fig. 9). At the SP location, roots of both varieties contained the highest values (0.28 and 0.21 mg/kg for Futura 75 and Tisza). All other tissues/organs contained less than 0.20 mg/kg of Cd, the lowest concentration was detected in seeds and female bark of Tisza (0.07 mg/kg in both). At the MP location, inflorescences of Tisza exhibited the highest value of Cd with 0.28 mg/kg, followed by roots of Tisza (0.27 mg/kg) and Futura 75 (0.22 mg/kg). Woody stem core of both varieties contained 0.10 mg/kg or less. Higher contents of Cd were measured at the HP location, where soil concentration was 6.6 mg/kg. Ranking of hemp organs at this location is as follows: Futura 75 inflorescences (1.66 mg/kg) > Futura 75 roots (0.92 mg/kg) > Futura 75 seeds (0.81 mg/kg) > Tisza roots (0.73 mg/kg) > Tisza roots (0.52 mg/kg) > Futura 75 stem bark (0.51 mg/kg). Other tissues/organs contained less than 0.50 mg/kg with Tisza female stem bark and Tisza female woody core having the lowest concentration (0.21 mg/kg). On average, hemp from the HP location contained the highest amount of Cd (0.58 mg/kg), where the content of Cd in hemp from MP (0.16 mg/kg) and from SP (0.14 mg/kg) did not differ markedly. The variety Futura 75 contained 0.39 mg/kg of Pb and the variety Tisza 1.8 times lower value. Regarding plant tissues/organs, the concentration gradient follows inflorescences (0.50 mg/kg) > roots (0.44 mg/kg) > monoecious stem bark (0.28 mg/kg) > seed (0.27 mg/kg) > male stem bark (0.26 mg/kg) > monoecious woody stem core (0.18 mg/kg) > female stem bark (0.14 mg/kg) > male woody stem core (0.14mg/kg) > female woody stem core (0.12 mg/kg).

Cd is one of the most studied elements in *Cannabis sativa* L. phytoremediation experiments. Di Candilo et al. (2004) noticed Cd uptake of up to 2.56 mg/kg by hemp roots grown in artificially polluted soil with 100 mg/kg of cadmium. Their plants were carefully uprooted meaning that fine roots were also analyzed. That is probably the reason for obtaining almost 6 times higher concentration than in our study, where only tap root was sampled. Fine roots could contain more than 100 times higher Cd content than tap roots (our unpublished results from pot experiments). Many other experiments were performed in pots in controlled environments (e.g., Citterio et al. 2003; Linger et al. 2005; Shi et al. 2009; Shi et al. 2012). In this kind of experiment, concentrations in different plant parts were very high, e.g., roots exceeded values over 1000 mg/kg, leaves showed up to 150 mg/kg, and stems up to 100 mg/kg. Generally, the content of Cd in the soil in pot experiments was also very high (up to 200 mg/kg) mostly due to the fact that Cd was artificially added and high contents were easy to obtain. These studies did not analyze the content of Cd in seed, because plants were sampled before seed maturity. This means that no biomass dilution effects due to longer growing period were present, explaining the higher content of Cd in hemp parts as well. Among various heavy metals, Cd was shown to be one of the most phytotoxic heavy metals (Prasad 1995; Salt et al. 1995). However, the mentioned studies showed that hemp is highly tolerant of high Cd presence in soil.

On the other hand, lower concentrations of Cd are seen in experiments performed in situ at polluted locations, similar to our study. Angelova et al. (2004), whose polluted soil contained a 2 times higher amount of Cd than in our research, detected the highest concentration in flowers (1.22 mg/kg) which corroborates the observation from our study, yet we determined lower values. They measured 1.00 mg/kg of Cd in seeds, which is 3.7 times higher than in our study. The same seed concentration (1.00 mg/kg) was also observed by Linger et al. (2002), although their experimental soil was very abundantly polluted with Cd (102 mg/kg). Mihoc et al. (2012) found up to 4.0 mg/kg of Cd in the seed of hemp grown in Cd-polluted gleyed chernozem. Regarding the EFSA Panel on Contaminants in the Food Chain, a tolerable weekly intake of Cd is 2.5 μg/kg body weight aiming to ensure sufficient protection for all kinds of consumers (Scientific report of EFSA, European Food Safety Authority 2012). For average adults (approx. 70 kg of weight), up to 100 g of seed from the HP location could be consumed daily to not exceed these EFSA guidelines.

Oeko-tex Standard 100 (Oeko-Tex-Initiative 2021) declares that the total content of Cd must be under 40 mg/kg, but the extractable concentration should not exceed 0.1 mg/kg. In this experiment, average concentration of Cd in stem bark was 0.23 mg/kg, which is way below the Oeko-tex Standard 100 (Oeko-Tex-Initiative 2021) limit for total Cd. It is expected that extractable Cd would also not exceed the Standard’s threshold since extractable HM concentrations are extremely low (Sungur and Gülmez 2015). Linger et al. (2002) determined a higher concentration of Cd in their fibers (up to 0.85 mg/kg). Nevertheless, this amount of Cd did not affect fiber quantity and quality, meaning that, very likely, fibers from our study also maintain bundle fineness and strength regardless of Cd presence. This could be useful information if fibers were used for technical, non-textile products (fabrics, insulation, building, composite material, etc.).

**Fig. 10:**
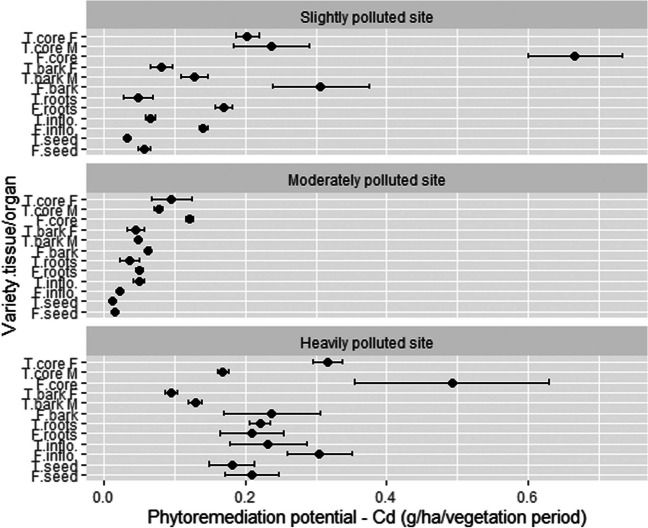
The phytoremediation potential for Cd of different hemp tissues/organs. T, Tisza; F, Futura 75; F, female; M, male; inflo, inflorescences; *n* = 3

Overall, the concentration of Cd was very low in all organs/tissues; therefore, the yield of these organs/tissues had a tremendous impact on the phytoremediation potential (Fig. 10). As expected, stem woody core contributed the majority to PP at each location. At SP, woody cores of Futura 75 and Tisza reached 0.67 and 0.44 (summed for male and female) g Cd/ha, respectively. Some higher PP was also exhibited by Futura 75 bark (0.31 g Cd/ha), whereas other organs/tissues showed PP lower than 0.21 g Cd/ha, the lowest by Tisza seed (0.03 mg Cd/ha). Futura 75 (0.49 g Cd/ha) and Tisza (0.48 g Cd/ha) woody cores also showed the best PP in the HP location, followed by inflorescences (0.27 g Cd/ha), bark (0.23 g Cd/ha), and roots (0.22 g Cd/ha). At the MP location, again woody cores of Tisza (0.17 g Cd/ha) and Futura 75 (0.12 g Cd/ha) stand out. Seeds achieved the lowest PP with 0.01 g Cd/ha. On average, PP by organs/tissues decrease in the order: woody core (0.40 g Cd/ha) > bark (0.19 g Cd/ha) > inflorescences (0.14 g Cd/ha) > roots (0.12 g Cd/ha) > seed (0.08 g Cd/ha). Cd uptake by hemp was most efficient at HP (0.23 g Cd/ha), intermediately successful at SP (0.18 g Cd/ha), and least feasible at MP (0.05 g Cd/ha). Futura 75 accumulated almost 2 times higher amount of Cd (1.34 g /ha) compared to Tisza (0.80 g/ha) at SP (average 1.07 g/ha), where Tisza (0.36 g/ha) outperformed Futura 75 (0.27 g/ha) at MP (average 0.32 g/ha). A smaller difference was observed at HP; average was 1.40 g/ha (1.45 g/ha for Futura 75 and 1.34 g/ha for Tisza).

Linger et al. (2002, 2005) reported extraction of 126 g and 830 g of Cd per ha in one vegetation period by hemp plants. These are much higher values compared to the outcome of our study. However, they used a different approach to determine PP; this is calculated by using the PP for one plant in their experiment and then extrapolating it to the theoretical number of plants per hectare area. This approach is less accurate and reliable, since the number of plants per hectare can be very variable due to self-thinning and other growing conditions (van der Werf et al. 1995) and hemp is known as a highly heterogenic species (Fike 2016), meaning that individual plants can differ greatly. A similar assessment of PP was done by Kos and Leštan (2003), Kos et al. (2003), and De Vos et al. (2022), who took literature data for the assessment of hemp biomass yield and calculated PP by multiplying with tissue concentration obtained in their researches. The first two studies determined PP up to 96 g/ha when using chelating agents [S,S]-EDDS or EDTA, and De Vos et al. (2022) determined up to 18 g/ha. In our experiment, growing conditions heavily impacted plants’ growth, meaning that yields of different hemp parts were very different from yields in some literature data. In any case, hemp plants in our trials exhibited very low efficiency for Cd soil extraction and were not able to accumulate significant quantities. This fact identified hemp as an excluder for Cd-contaminated sites, since excluders are plants that can grow in heavy metal–polluted soils without accumulating larger amounts (Baker 1981). We also noticed that absorbed Cd was not retained in the roots but was rather translocated to the above-ground parts. This observation does not comply with the results of Shi and Cai (2009) and Shi et al. (2009, 2012). However, in other field experiments with hemp (Linger et al. 2002; Angelova et al. 2004), a high content of Cd in above-ground biomass compared to roots was also observed, as in our study.

### General discussion

Biological, technological, and economical components are the three elements that decide whether using plants for soil remediation is exploitable and cost efficient. The relationship among these components is proportionate and when one shows weaknesses, other components must be stronger to take over in such a balanced scheme (Griga and Bjelková 2013). The main constraint for using hemp in the phytoremediation process is its biological component. Although some studies address hemp as a hyperaccumulator for different toxic trace metals such as lead, cadmium, magnesium, copper, chromium, and cobalt (Girdhar et al. 2014; Zielonka et al. 2020; Praspaliauskas et al. 2020), our study showed low ability of hemp to accumulate Pb (up to 10.6 mg/kg), Zn (up to 151.9 mg/kg), and Cd (up to 1.66 mg/kg). Real hyperaccumulators for the listed three metals are capable of accumulating 1000 mg/kg of Pb, 10,000 mg/kg of Zn, and 100 mg/kg of Cd dry weight (DW) in their aerial parts (Baker and Brooks 1989).

However, hemp still offers a high potential to be used for phytoremediation purposes not only due to its fast growth in a variety of climates and soil types, its natural resistance to many biotic and abiotic stresses, and its well-known technique of cultivation (Small 2015; Fike 2016; Amaducci et al. 2015; Adesina et al. 2020; Żuk-Gołaszewska and Gołaszewski 2020), but also mostly due to its high above-ground biomass production (Kumar et al. 2017b; Todde et al. 2022). This fact leads to another very important aspect of why hemp could be grown in heavy metal–polluted soil; this is its multipurpose usage and extensive industrial utilization of all plant parts, even when they contain high amounts of heavy metals. Fibers and seeds show the highest market values with combined revenue of 3117–4898 €/ha per year (Moscariello et al. 2021). However, our study revealed that seeds from locations HP and MP contained a content of Pb that was too high to be used in the food industry. On the other hand, stem bark with fibers did not contain too high values of any heavy metal investigated, meaning that using it for textiles would be safe. Nevertheless, numerous alternatives for using contaminated seeds and stems for many kinds of industrial products with added value exist and these products would not pose a health risk, e.g., contaminated fibers can be used as tech products (cordage, canvas, biocomposites, bioplastics for non-food uses, etc.). Hurd with increased concentration of heavy metals could be incorporated in construction and building materials (e.g., fiberboard and hempcrete) or for the production of thermal insulators. Combined materials could be built out of contaminated material, where the fibers are embedded in polymers and could not be set free. Other non-consumable and non-textile applications are in the pulp-and-paper industry, furniture industry, and chemical industry (paints, oils, and varnishes) (Crini et al. 2020; Rehman et al. 2021). Another very promising use of hemp is the production of bioenergy. A critical review on the simultaneous usage of hemp for phytoremediation and bioenergy production (biodiesel, biogas, bioethanol, and soil biofuels) was recently provided by Rheay et al. (2020), who concluded that hemp is a suitable alternative crop that can be applied for phytoremediation purposes and feasibility of this operation could be justified by generating profit from its dual purpose use. Todde et al. (2022) upgraded conclusions about the feasibility of combining phytoremediation technologies with the energy valorization of contaminated hemp biomass by highlighting the problem of the lack of clear regulatory framework in this area. However, all approaches to alternative hemp utilization after phytoremediation considered hemp as a “resource” rather than “waste,” thus fulfilling the multifaceted potential of this crop and proving that it fits well in the holistic circular bioeconomy model (Wu et al. 2019).

Our on-site experiment on agricultural land with real soil pollution problems confirmed observations in other studies (Griga and Bjelkova 2013; Rheay et al. 2020; Wu et al. 2019), that the results of greenhouse pot experiments cannot be directly extrapolated to field trials, where complex environment and unpredictable weather conditions significantly influence the final outcome. For example, a hail storm severely damaged plants at the HP location in this study, thus significantly decreasing the phytoremediation potential of hemp in this location. Therefore, implementation of hemp in real-world phytoremediation projects should be analyzed on a case-by-case basis in order to determine the most efficient hemp genetics (variety) and most suitable agrotechnique. Ideally, assessment should be constructed on the basis of the evaluation of growing hemp in polluted locations over multiple growing seasons.

## Conclusion

This study was performed on site on agricultural land in real polluted soils using approved hemp varieties, where all needed data (including biomass yield of various plant parts) were obtained in order to draw solid conclusions about hemp’s suitability for use in the remediation of polluted soils in the Celje valley. Our study showed that uptake of Pb, Zn, and Cd in different hemp organs/tissues was low overall. Test locations with different contents of heavy metals in soil had a significant influence on the metal uptake, where, as expected, plants at the HP location accumulated higher values of Pb, Zn, and Cd in their organs/tissues compared to those at the MP and SP locations. The results showed that phytoremediation potential from this investigation was too low to enable us to consider hemp as a serious remediation agent for cleaning up polluted soil in the Celje valley. Nevertheless, the advantages of growing hemp are its multipurpose utilization and potential to produce economic benefits.
